# Supplementary material for: Recruited macrophages that colonize the post-inflammatory peritoneal niche convert into functionally divergent resident cells
Source: Nat Commun. 2021 Mar 19;12:1770. doi: 10.1038/s41467-021-21778-0 (PMC7979918; doi:10.1038/s41467-021-21778-0)
Supplement: Supplementary file 1 — Supplementary Information [file 41467_2021_21778_MOESM1_ESM.pdf]

Supplementary Information

Recruited macrophages that colonise the post-inflammatory peritoneal niche convert into functionally divergent resident cells

Louwe et al.

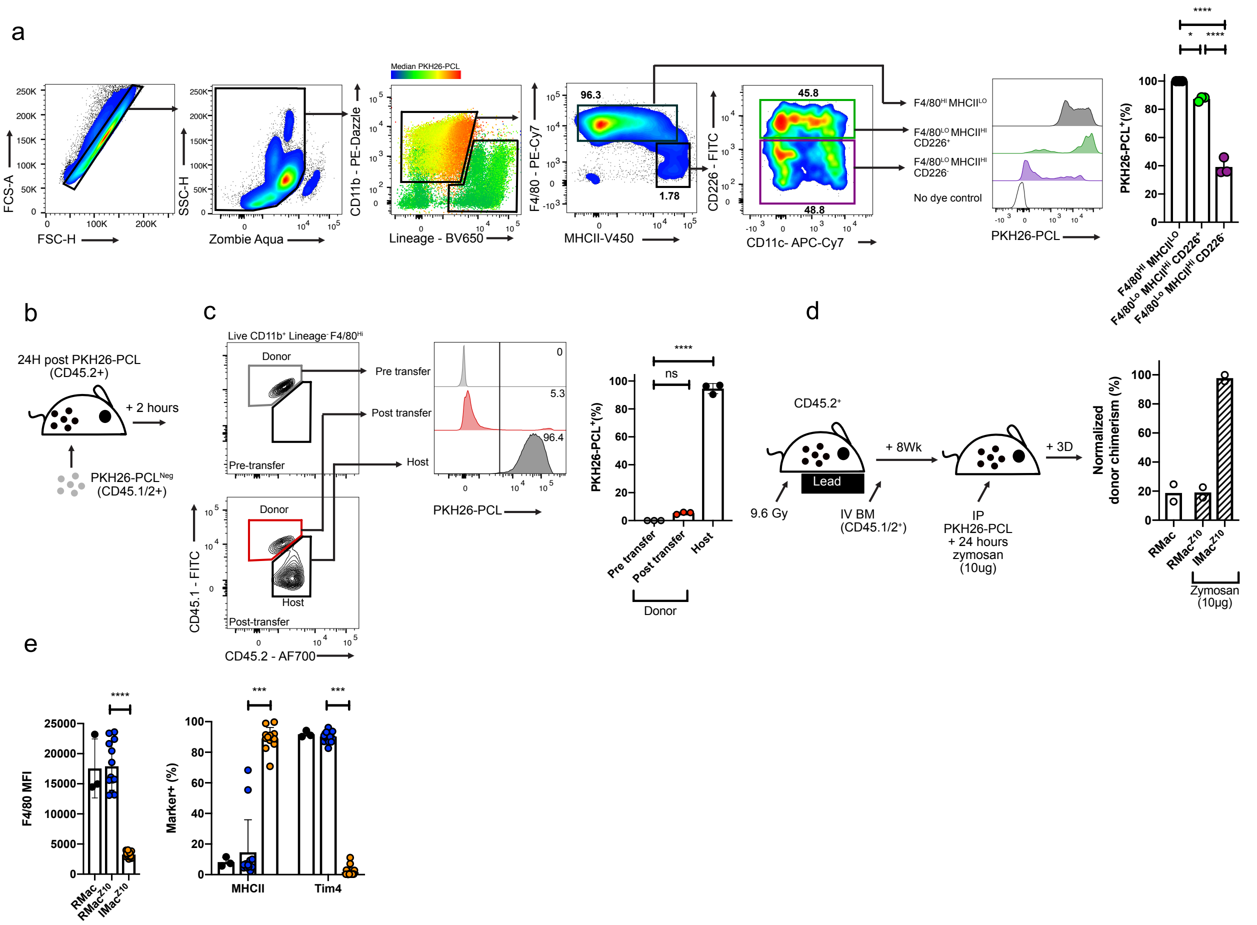

Supplementary Figure 1. A toolbox to investigate inflammatory macrophage fate

(a) Dye labelling efficiency of F4/80^{HI} MHCII^{LO} resident macrophages (grey) and F4/80^{LO}MHCII^{HI} CD226^{+} small peritoneal macrophages (green) or CD226^{-} DCs or immature macrophages (purple) 24hrs after intraperitoneal administration of PKH26-PCL (n=3). *p<0.05, **p<0.01, ****p<0.0001 by determined one-way ANOVA with Tukey's multiple comparisons test. This sequential gating strategy to identify peritoneal CD11b^{+} Lineage^{-} myeloid cells has been used throughout the manuscript.

(b) Experimental scheme for the adoptive transfer of unlabelled CD45.1/2^{+} peritoneal exudate cells (PEC) into the peritoneal cavity of CD45.2^{+} mice injected with PKH26-PCL intraperitoneally 24hrs prior.

(c) Representative PKH26-PCL labelling and quantification of donor F4/80^{hi} macrophages prior to transfer (top) and 2hrs post transfer (red; n=3) compared to recipient F4/80^{Hi} macrophages (black). p<0.0001 (****) determined by one-way ANOVA with Tukey's multiple comparisons test.

(d) Non-host chimerism of F4/80^{HI} PKH26-PCL^{HI} RMac in the naïve peritoneal cavity (white bar) and F4/80^{HI} PKH26-PCL^{HI} RMac^{Z10} and PKH26-PCL^{LO} F4/80^{INT} IMac^{Z10} 3d post 10μg zymosan (hatched bars). Dye injection given 8wks after irradiation and zymosan injection given 24hrs thereafter.

(e) Expression of F4/80, MHCII and Tim4 by RMac (black, n=3), RMac^{Z10}(blue, n=13) and IMac^{Z10} (orange, n=13), 3 days post 10μg zymosan. F4/80MFI: $p<0.0001$ (****), MHCII/Tim4 both $p=0.0002$(***) determined one-way ANOVA with Dunnet's multiple comparisons test for each marker individually, followed by Bonferroni adjustment.

For all experiments, data are presented as mean ± standard deviation with each symbol representing an individual animal. All data were pooled from at least 2 independent experiments, except for **(a,c,d)** which are from single experiments.

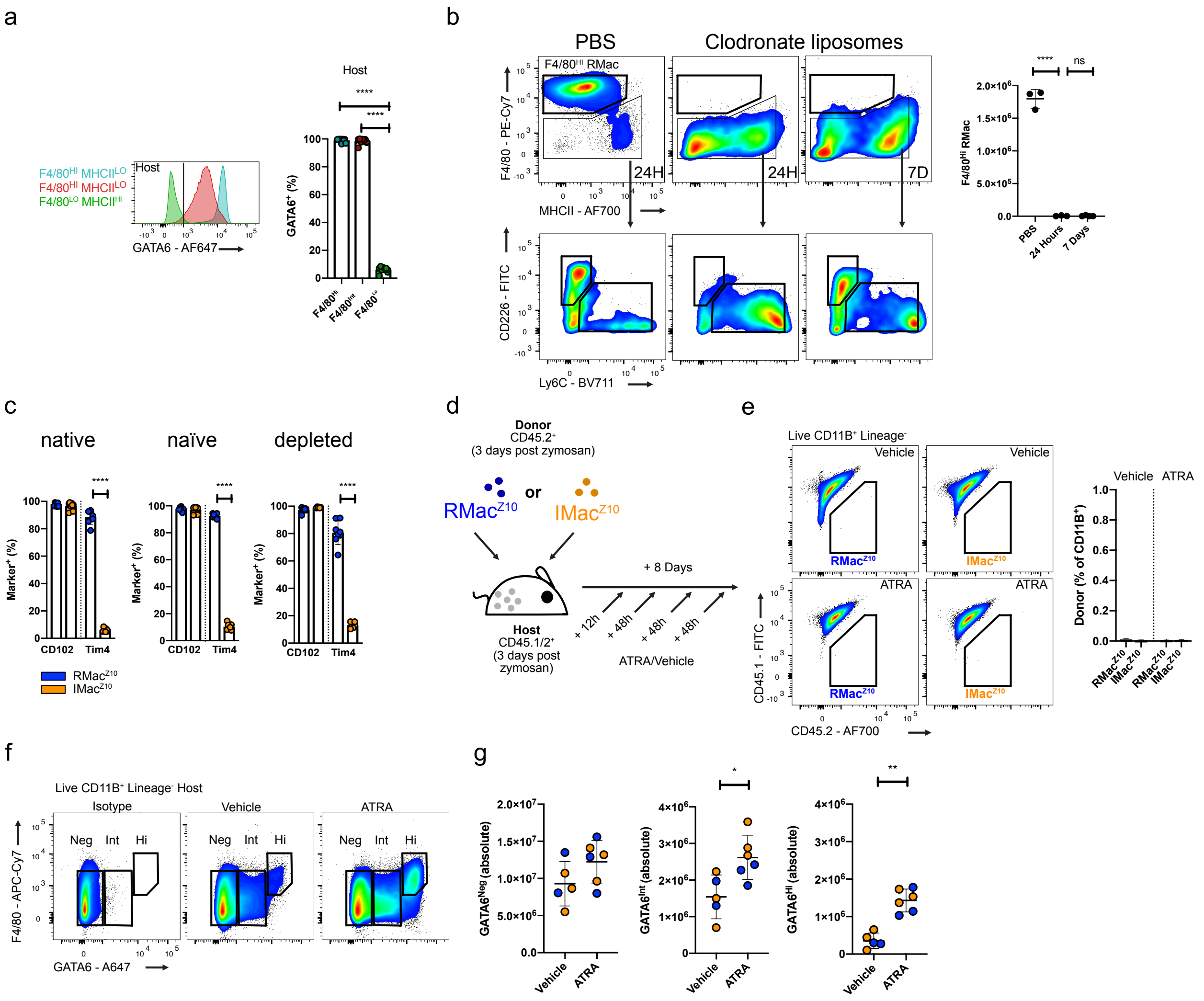

Supplementary Figure 2. Competition mediates inflammatory macrophage survival and phenotype during early resolution

(a) Proportion of host macrophage subsets that are GATA6^{+} 11d post zymosan (8d post cell transfer; n=9) ****p<0.0001 determined by one-way ANOVA with Tukey's multiple comparisons test.

(b) Representative dot-plots gated on CD11b^{+} cells and absolute number of F4/80^{Hi} resident macrophages (black) 24hrs after intraperitoneal injection of PBS (n=3) or clodronate liposomes (n=3) and 7d post clodronate liposome injection (n=4). p<0.0001 (****) determined by one-way ANOVA and Dunnet's multiple comparisons test.

(c) Proportion of donor RMac^{Z10} and IMac^{Z10}that are CD102^{+} and Tim4^{+} 8d after transfer into mirroring native (n= 7, 8), naïve (both n=7) or clodronate depleted recipients (n= 8,6). For each condition: p<0.0001 (****) determined by one-way ANOVA and Sidak's multiple comparisons test.

(d) Experimental scheme for the adoptive transfer of $RMac^{Z10}$ (blue) or $IMac^{Z10}$ (orange) sourced from CD45.2 mice 3d after injection of 10μg zymosan into mirroring inflamed CD45.1/2 recipient mice followed by ATRA or vehicle treatment regimen.

(e) Proportion of $CD11b^{+}Lineage^{-}$ cells that are of donor origin after indicated treatment.

(f) Representative expression of F4/80 and GATA6 by host myeloid cells after indicated treatment.

(g) Absolute number of $GATA6^{Neg}$, $GATA6^{Neg}$ and $GATA6^{Hi}$ macrophages (left to right) after vehicle treatment or ATRA treatment (both n=5).$GATA6^{Int}$: p=0.016 (*),$GATA6^{Int}$: p<0.0001 (****), determined by student's t test. Datapoints coloured orange are $IMac^{Z10}$ recipients, datapoints coloured in blue are $RMac^{Z10}$ recipient mice.

For all experiments, data are presented as mean ± standard deviation with each symbol representing an individual animal. Data presented in (a,c) were pooled from 2 independent experiments, other data was from a single experiment.

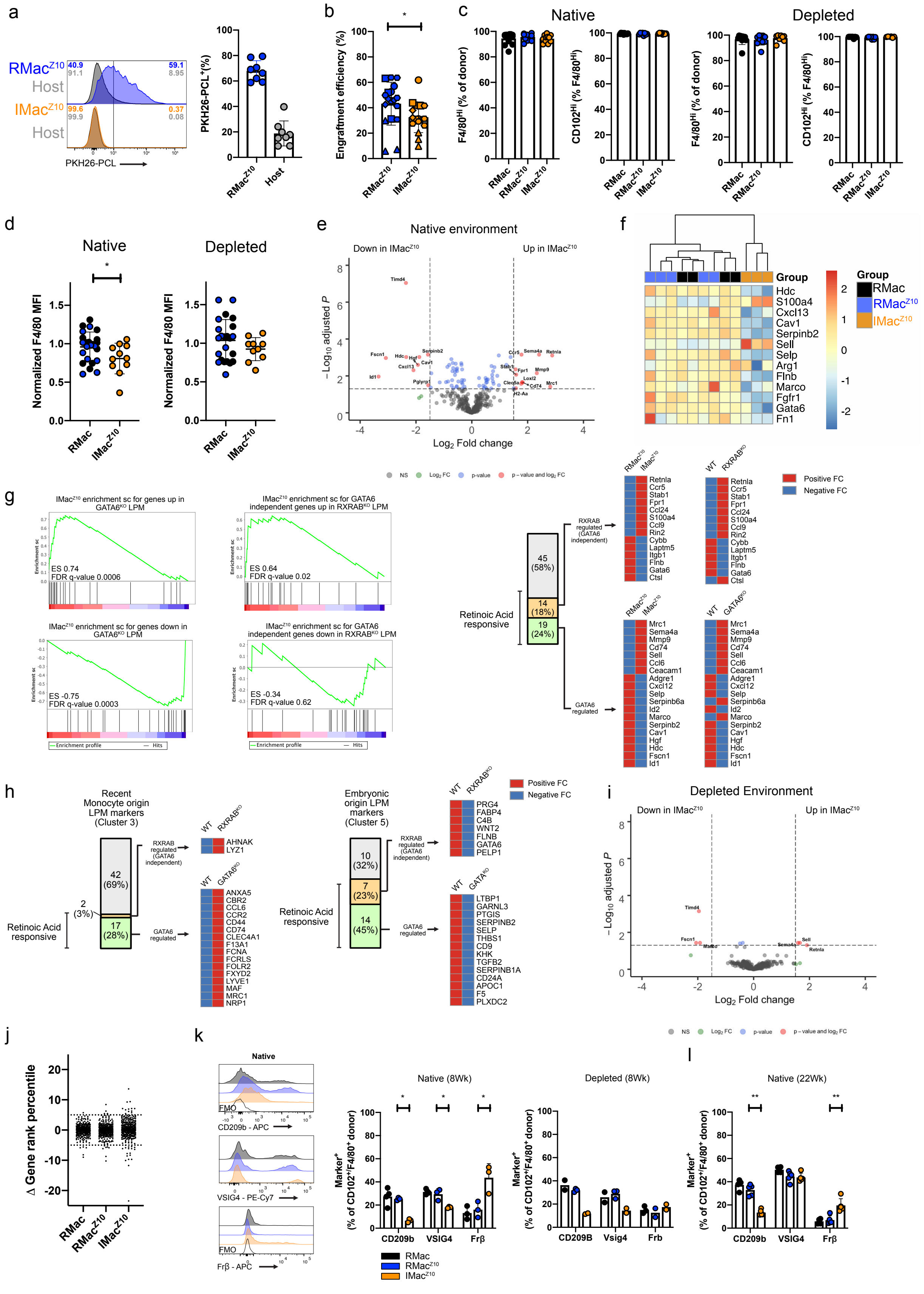

a
RMacZ10
Host
IMacZ10
Host
PKH26-PCL
PKH26-PCL+(%)
RMacZ10
Host
b
Engraftment efficiency (%)
RMacZ10
IMacZ10
c
Native
Depleted
F4/80Hi (% of donor)
CD102Hi (% F4/80Hi)
RMac
RMacZ10
IMacZ10
d
Native
Depleted
Normalized F4/80 MFI
RMac
IMacZ10
e
Native environment
Down in IMacZ10
Up in IMacZ10
– Log10 adjusted P
Log2 Fold change
NS
Log2 FC
p-value
p – value and log2 FC
f
Group
Hdc
S100a4
Cxcl13
Cav1
Serpinb2
Sell
Selp
Arg1
Flnb
Marco
Fgfr1
Gata6
Fn1
Group
RMac
RMacZ10
IMacZ10
g
IMacZ10 enrichment sc for genes up in GATA6KO LPM
ES 0.74
FDR q-value 0.0006
IMacZ10 enrichment sc for GATA6 independent genes up in RXRABKO LPM
ES 0.64
FDR q-value 0.02
IMacZ10 enrichment sc for genes down in GATA6KO LPM
ES -0.75
FDR q-value 0.0003
IMacZ10 enrichment sc for GATA6 independent genes down in RXRABKO LPM
ES -0.34
FDR q-value 0.62
Enrichment profile
Hits
Retinoic Acid responsive
45 (58%)
14 (18%)
19 (24%)
RXRAB regulated (GATA6 independent)
GATA6 regulated
RMacZ10
IMacZ10
WT
RXRABKO
GATA6KO
Positive FC
Negative FC
Retnla
Ccr5
Stab1
Fpr1
Ccl24
S100a4
Ccl9
Rin2
Cybb
Laptm5
Itgb1
Flnb
Gata6
Ctsl
Mrc1
Sema4a
Mmp9
Cd74
Sell
Ccl6
Ceacam1
Adgre1
Cxcl12
Selp
Serpinb6a
Id2
Marco
Serpinb2
Cav1
Hgf
Hdc
Fscn1
Id1
h
Recent Monocyte origin LPM markers (Cluster 3)
42 (69%)
2 (3%)
17 (28%)
Retinoic Acid responsive
RXRAB regulated (GATA6 independent)
GATA6 regulated
WT
RXRABKO
GATA6KO
AHNAK
LYZ1
ANXA5
CBR2
CCL6
CCR2
CD44
CD74
CLEC4A1
F13A1
FCNA
FCRLS
FOLR2
FXYD2
LYVE1
MAF
MRC1
NRP1
Embryonic origin LPM markers (Cluster 5)
10 (32%)
7 (23%)
14 (45%)
Positive FC
Negative FC
PRG4
FABP4
C4B
WNT2
FLNB
GATA6
PELP1
LTBP1
GARNL3
PTGIS
SERPINB2
SELP
THBS1
CD9
KHK
TGFB2
SERPINB1A
CD24A
APOC1
F5
PLXDC2
i
Depleted Environment
Down in IMacZ10
Up in IMacZ10
– Log10 adjusted P
Log2 Fold change
NS
Log2 FC
p-value
p – value and log2 FC
j
Δ Gene rank percentile
RMac
RMacZ10
IMacZ10
k
Native
FMO
CD209b - APC
VSIG4 - PE-Cy7
Frβ - APC
Native (8Wk)
Depleted (8Wk)
Marker+ (% of CD102+F4/80+ donor)
CD209b
VSIG4
Frβ
CD209B
Vsig4
Frb
RMac
RMacZ10
IMacZ10
l
Native (22Wk)
CD209b
VSIG4
Frβ

Supplementary Figure 3. Colonizing inflammatory macrophages are long lived but retain cell intrinsic and environment dependent transcriptional and phenotypic differences

(a) Proportion of donor CD45.2^{+} F4/80^{Hi} RMac^{Z10}and host CD45.1/2^{+} F4/80^{Hi} macrophages that are PKH26-PCL^{+} 8wks post transfer (n=8).

(b) Pooled engraftment efficiency of RMac^{Z10} and IMac^{Z10} 8wks post transfer into native recipients. Includes data presented in Figure 2b and Figure 4b. Each symbol refers to an experimental run. p=0.043 (*) determined by student's t test.

(c) Fraction of donor RMac, RMac^{Z10} and IMac^{Z10} that are F4/80^{Hi} and the proportion of which are CD102^{Hi} after transfer into native (left; n= 12,11,11) and depleted recipients (right; n = 10,8,8).

(d) Normalized F4/80 MFI on donor RMac(black), RMac^{Z10} (blue) and IMac^{Z10} (orange) after transfer into native (left; n=12,11,11) or depleted (right; n=10,10,8) recipients. F4/80 MFI is normalized to mean F4/80 MFI of RMac. p=0.042 (*) determined by student's t test.

(e) Volcano plot of gene expression of IMac^{Z10} relative to RMac^{Z10} 8wks post transfer into native recipients. Adjusted p-values were calculated using the Benjamini-Hochberg methodology.

(f) Heatmap highlighting the subset of peritoneal macrophage identity genes included in the NanoString panel and their expression by donor RMac, RMac^{Z10} and IMac^{Z10} 8wks post transfer into native recipients.

(g) GSEA of mrNA in RMac^{Z10}and IMac^{Z10} against genes up and downregulated genes in GATA6^{KO} LPM or RXRAB^{KO}LPM. To the right, proportion of differentially expressed genes between IMac^{Z10} and RMac^{Z10} that are regulated by GATA6 or RXRAB and their transcriptional directionality. On the right, transcriptional directionality of the same genes in GATA6^{KO}/RXRAB^{KO} LPM relative to WT.

(h) Proportion of highly expressed single cell cluster genes that are regulated by GATA6/RXRAB in cluster 3 and 5 as described by Bain et al[15] and the transcriptional directionality of these genes in GATA6^{KO}/RXRAB^{KO} LPM relative to WT.

(i) Volcano plot of gene expression of IMac^{Z10} relative to RMac^{Z10} 8wks post transfer into depleted recipients. Adjusted p-values were calculated using the Benjamini-Hochberg methodology.

(j) Change in expression rank for each detected gene following transfer inter into native or clodronate-depleted recipients (ΔGene rank percentile = Percentile rank in native environment – Percentile rank in clodronate-depleted environment). Dashed lines denote percentile change of 5%.

(k) Expression of markers of interest by CD102^{+} or F4/80^{+} donor RMac (black) RMac^{Z10} (blue), IMac^{Z10} (orange) 8wks post transfer into native (n=4,3,3) and depleted recipients (n=2,3,2). Data obtained from a single experiment. *p<0.05 determined by one way ANOVA and Dunnet's multiple comparisons test for each marker individually, followed by Bonferroni adjustment.

(l) Expression of markers of interest by CD102$^+$ or F4/80$^+$ donor RMac(black; n=4) RMac^{Z10} (blue; n=5), IMac^{Z10} (orange; n=5) 22wks post transfer into native recipients. **p<0.01 determined by one way Anova and Dunnet's multiple comparisons test for each marker individually, followed by Bonferroni adjustment.

For all experiments, data are presented as mean ± standard deviation with each symbol representing an individual animal. All data were pooled from at least 2 independent experiments, except for **(k)** which is from a single experiments.

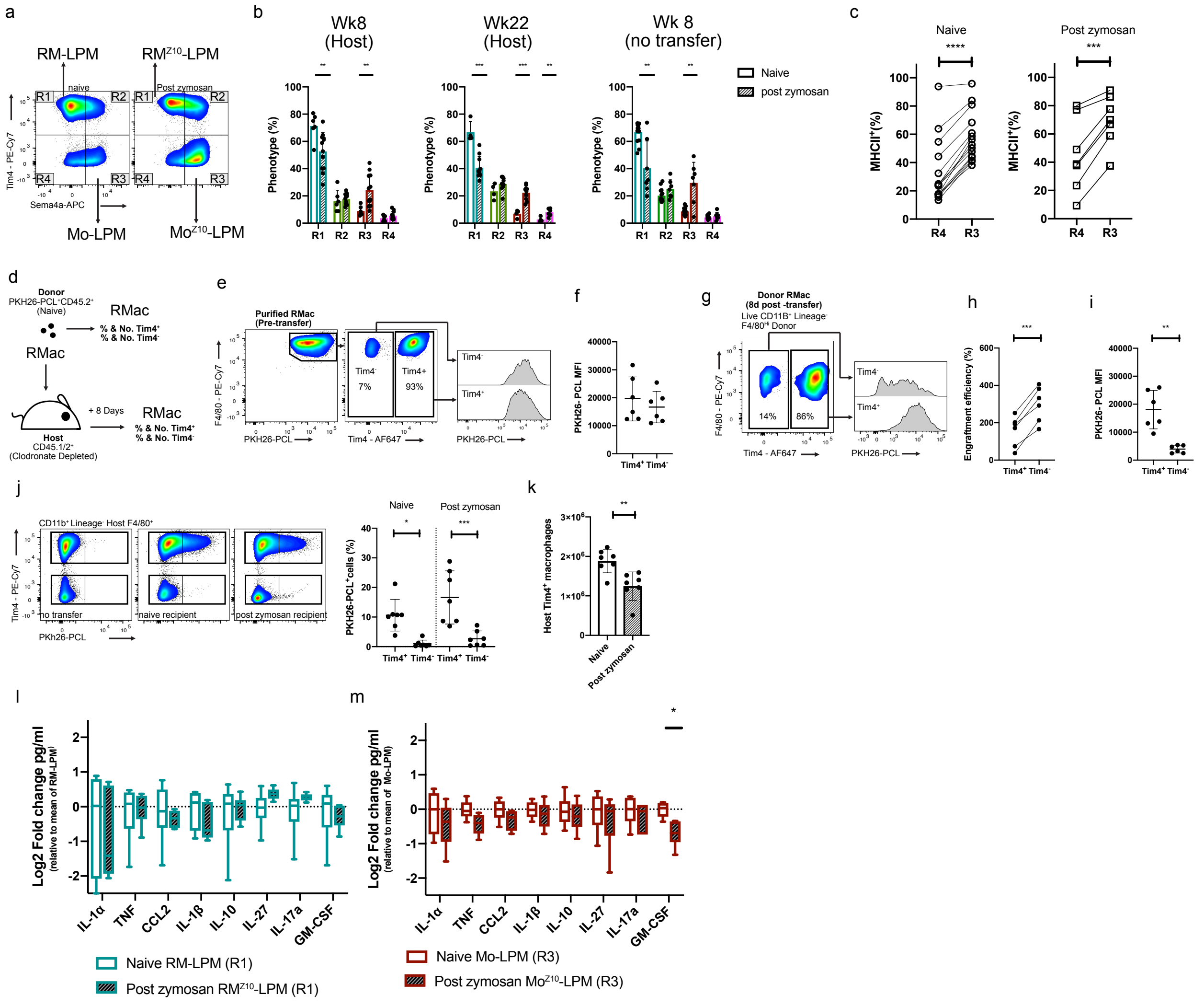

Supplementary Figure 4. Monocyte derived LPM are functionally distinct from embryonically seeded LPM

(a) Expression of Tim4 and Sema4a by LPM from naïve mice or 8wks post zymosan injection.

(b) Proportion of host LPM with Sema4a^{LO}Tim4^{+}(R1), Sema4a^{HI}Tim4^{+}(R2), Sema4a^{Hi}Tim4^{-} (R3) or Sema4a^{Lo}Tim4^{-} (R4) phenotype 8wks post zymosan (6 naïve v 12 zymosan treated) and 22wks post zymosan 4 naïve v 10 zymosan treated) compared to naïve control. Right, proportion of LPM with Sema4a^{LO}Tim4^{+}(R1), Sema4a^{HI}Tim4^{+}(R2), Sema4a^{Hi}Tim4^{-} (R3) or Sema4a^{Lo}Tim4^{-} (R4) phenotype 8wks post zymosan (15 naïve v 8 zymosan treated). ***p<0.001**p<0.01, repeated student's t test with Holm-Sidak correction.

(c) Proportion of Sema4a^{Lo}Tim4^{-} (R4) and Sema4a^{Hi}Tim4^{-} (R3) that are MHCII^{+} in naïve (n=15) and zymosan treated (n=7) mice. Naïve: p<0.0001 (****), post zymosan: p=0.0007(***), paired student's t test.

(d) Experimental scheme for transfer of RMac (black) sourced from naïve CD45.2 mice into mirroring clodronate-depleted CD45.1/2 recipient mice.

(e) Expression of Tim4 and PKH26-PCL labelling on donor RMac following purification and prior to transfer.

(f) MFI of PKH26-PCL labelling on purified Tim4+ or Tim4- donor RMac prior to transfer (n=6).

(g) Expression of Tim4 and PKH26-PCL labelling on donor RMac following 8 days following transfer into clodronate-depleted recipients.

(h) Engraftment efficiency of $Tim4^{+}/Tim4^{-}$ RMac, 8 days following transfer into clodronate-depleted recipients (n=6). p=0.0003 (***), paired student's t test.

(i) MFI of PKH26-PCL labelling on $Tim4^{+}$ or $Tim4^{-}$ donor, 8 days following transfer into clodronate-depleted recipients. p=0.0034 ***), paired student's t test.

(j) Fraction of naïve or post zymosan host $F4/80^{+}Tim4^{+/-}$ macrophages that are PKH26-PCL labelled 8d after receiving PKH26-PCL labelled RMac (n=7). *p<0.05***p<0.001, one way ANOVA with Sidak multiple comparison test.

(k) Number of host naïve or post zymosan host $Tim4^{+}$ macrophages 8d after receiving PKH26-PCL labelled RMac (n=7). p=0.0035 (**), student's t test.

(l) Analytes in culture media of RM-LPM or RM^{Z10}-LPM (both n=6), sourced from naïve or 8wks post zymosan mice, 14hrs after culture with LPS (1ng/ml). Results are shown as log2 fold change in mean pg/ml over mean RM-LPM. Box extends from the 25th to the 75th percentile, middle line denotes median. Whiskers denote minima and maxima.

(m) Analytes in culture media of Mo-LPM or Mo^{Z10}-LPM (both n=6), sourced from naïve or 8wks post zymosan mice, 14hrs after culture with LPS (1ng/ml). Results are shown as log2 fold change in mean pg/ml over mean Mo-LPM. Box extends from the 25th to the 75th percentile, middle line denotes median. Whiskers denote minima and maxima. *p<0.05, repeated student's t test with Holm-Sidak correction.

Data are presented as mean ± standard deviation with each symbol representing an individual animal. Data were pooled from at least 2 independent experiments.

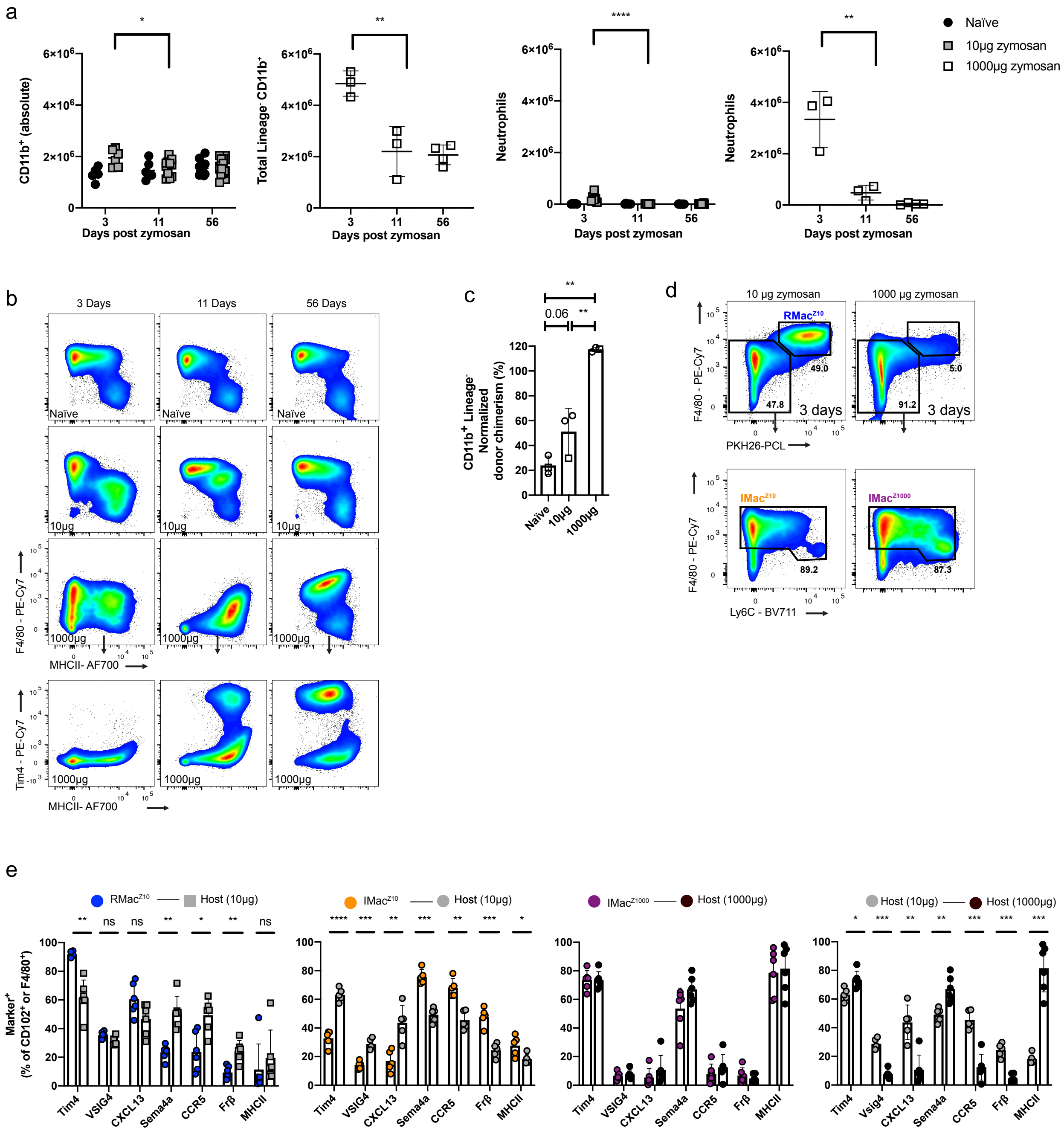

Supplementary Figure 5. Ontogeny does not dictate monocyte phenotype after severe peritonitis. (a) Absolute number of CD11b⁺ myeloid cells and neutrophils at indicated timepoints in naïve (black circle; n= 5,6,10), post low dose zymosan (grey square; n=6,11,20) and post high dose zymosan (white square; n=3,3,4). From left to right: p=0.043(*), p=0.0042(**), p<0.0001(****), p=0.0019(**). For naïve and low

dose determined by two-way ANOVA followed by post hoc Tukey test. For high dose determined by one-way ANOVA and post hoc Tukey test

(b) Representative expression of F4/80, MHCII and Tim4 by at indicated timepoints in naïve, 10µg or 1000µg zymosan treated mice.

(c) Non host chimerism of $CD11b^+$ myeloid cells 17d after indicated zymosan dose in tissue-protected BM chimeric mice (n=3/group). Zymosan treatment 8 (circle) or 26 (square) wks after irradiation. **p<0.01 determined by one-way ANOVA and Tukey's multiple comparisons test

(d) Representative expression of F4/80, Ly6C and PKH26-PCL labelling and identification of $F4/80^{HI}$ PKH26-PCL^{HI} resident macrophages ($RMac^{Z10}$) and PKh26-PCL^{LO} $F4/80^{INT}$ inflammatory macrophages 3d after 10µg ($IMac^{Z10}$) or 1000µg ($IMac^{Z1000}$) zymosan.

(e) Expression of markers of interest by $CD102^+$/$F4/80^+$ donor $RMac^{Z10}$(n=6) ,$IMac^{Z10}$ (n=5) or $IMac^{Z1000}$ (n=6) and their respective $CD102^+$/$F4/80^+$ host macrophages. *p<0.05 **p<0.01 ***p<0.001 ****p<0.0001 determined by repeated student's t test with Holm-Sidak correction.

For all experiments, data are presented as mean ± standard deviation with each symbol representing an individual animal. All data were pooled from at least 2 independent experiments except (a) where high dose datapoints where from a single experiment.

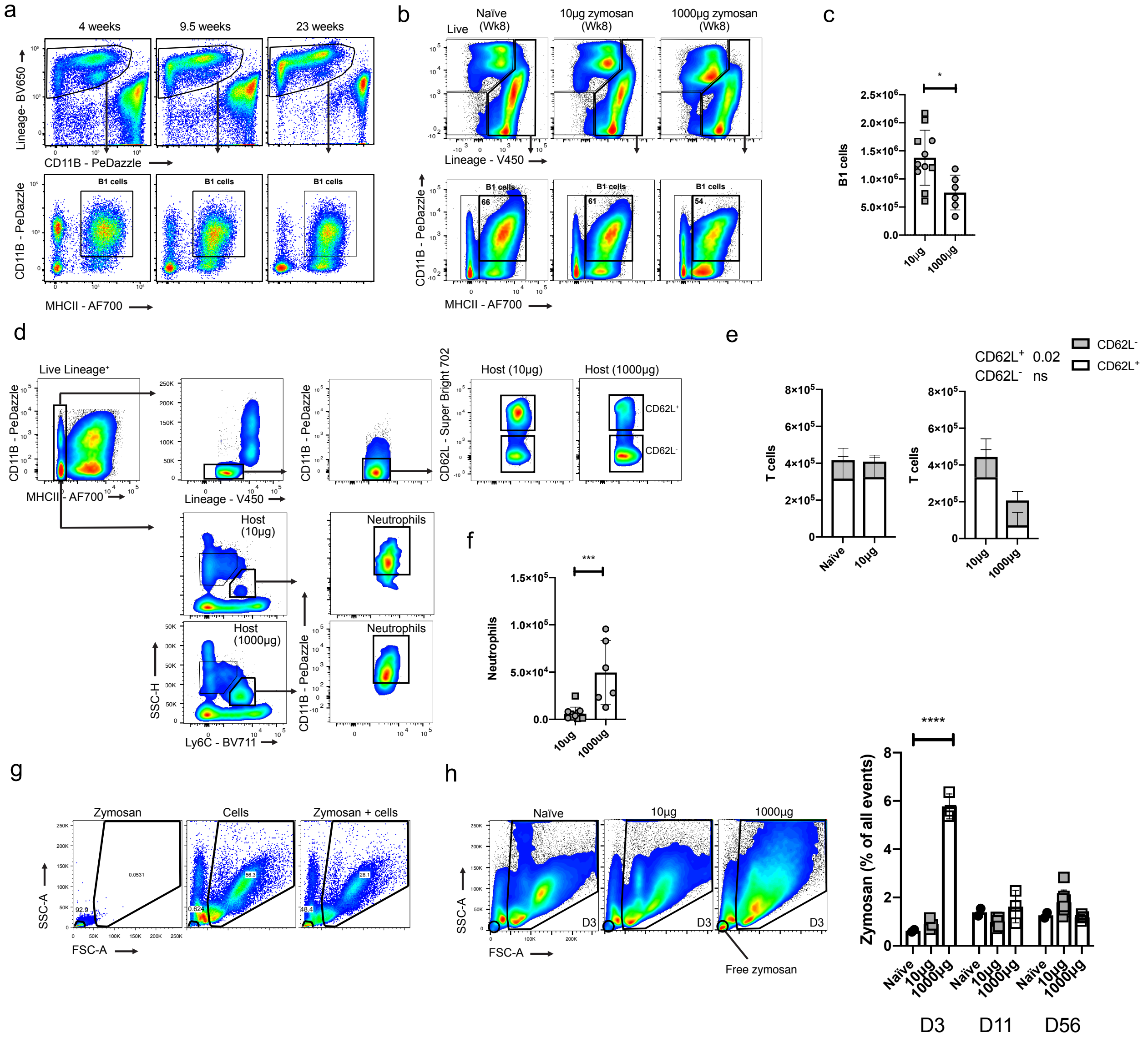

Supplementary Figure 6. Severe peritonitis leads to long-term perturbations of peritoneal immune cells.

(a) Gating strategy to identify peritoneal CD11b$^+$B1 cells in naïve female mice of indicated age.

(b) Gating strategy to identify peritoneal CD11b$^+$B1 in mice 8 weeks after indicated treatment.

(c) Absolute number of CD11b$^+$ B1 cells 8wks after 10µg of zymosan (n=11) or 1000µg zymosan (n=6) with transfer or IMac^{Z10}/RMac^{Z10} or IMac^{1000} at D3 respectively. p=0.014 (*) determined by student's t test.

(d) Representative gating strategy to identify T-cell subsets and Neutrophils.

(e) Absolute number of CD62L$^-$T cells (grey) and CD62L$^+$T cells (white) that are present in the cavity of naïve mice (n=7) or mice treated with 10µg of zymosan 8 wks prior(n=12) with transfer of RMac or IMac^{Z10}/RMac^{Z10} respectively. To the right, quantification of the same in mice treated with 10µg of

zymosan 8 wks prior (n=11) or 1000µg zymosan (n=6) with transfer or IMac^{Z10}/RMac^{Z10} or IMac^{1000} at D3 respectively. Statistical significance determined using repeated t test with Holm-Sidak correction.

(f) Absolute number of neutrophils 8 wks after 10µg of zymosan (n=11) or 1000µg zymosan (n=6) with transfer of IMac^{Z10}/RMac^{Z10} or IMac^{1000} at D3 respectively. p=0.0008 (***) determined by student's t test.

(g) Identification of zymosan particles on the basis of SSC-A/FCS-A relative to peritoneal cells.

(h) Proportion of events that are zymosan particles in naïve mice, mice treated with 10µg of zymosan and mice treated with 1000µg of zymosan at D3 (n=2,3,3 respectively), D11 (n=2,5,3 respectively) and D56 (n=2,8,4 respectively). p<0.0001(****) determined by two way ANOVA with Tukey's multiple comparisons test.

For all experiments, data are presented as mean ± standard deviation with each symbol representing an individual animal. All data were pooled from at least 2 independent experiments except (g,h) originate from a single experiment.

Supplementary Table 1: List of reagents used.

Reagent	Source	Catalogue #
Brefeldin A	Biolegend	420601
BSA	Sigma-Aldrich	A7906-100g
Casein	VWR	22544.292
Clodronate Liposomes	Liposoma	n/a
Corn Oil	Sigma-Aldrich	C8267-500ML
dPBS	Gibco/ThermoFisher	14190-094
EDTA 0.5M	Invitrogen	15574020
FCS	Gibco/ThermoFisher	10500-064
Folic Acid	Sigma-Aldrich	F8758-5G
FSC (LE confirmed in house)	GE-Healthcare	
HEPES 1M	Fisher Scientific	10041703
Intracellular Fixation and Permeabilization set	ThermoFisher	889-8824-00
L-Glutamine 200mM	Gibco/ThermoFisher	25030024
LEGENDplex mouse anti-virus	Biolegend	740622
LEGENDplex mouse inflammation	Biolegend	740446
LPS (O111:B4	Sigma-Aldrich	L2630-10MG
LPS (O127:B8)	Sigma-Aldrich	L3129-10MG
Macrophage SFM	Gibco/ThermoFisher	12065074
Monensin	Biolegend	420701
PC-BSA	2B Scientific	PC-1011-10
Pen/Strep	Gibco/ThermoFisher	15140122
Phrodo *E.coli* phagocytosis kit	Invitrogen/ThermoFisher	A10025
PKH26-PCL Cell Linker kit	Sigma-Aldrich	PKH26PCL-1KT
Recombinant murine CSF1	Peprotech	315-02
Retinoic Acid	Sigma-Aldrich	R2625-50MG
RPMI 1640	Gibco/ThermoFisher	21870076
RPMI 1640 no folic acid	Gibco/ThermoFisher	27016021
TMB	KPL sureblue reserve	5120-0081
TWEEN	Sigma-Aldrich	P1379
Zombie Aqua	Biolegend	423102
Zymosan A	Life technologies	Z4250-250MG

Supplementary Table 2: List of antibodies used

Antibody	Clone	Source	Fluorochrome	Catalogue #	Dilution
CD3	17A2	Biolegend	Biotin	100244	1:200
			PB	100214	1:200
CD11b	M1/70	Biolegend	PE-Dazzle	101256	1:400
CD11c	N418	Biolegend	APC-Cy7	117324	1:200
CD16/32	2.4G2	Biolegend	Purified	101320	1:200
CD19	6D5	Biolegend	Biotin	115504	1:200
			PB	115523	1:200
CD45.1	A20	Biolegend	FITC	110706	1:200
			AF700	110724	1:100
CD45.2	104	Biolegend	AF700	109822	1:200
CD102	3C4	Biolegend	FITC	105606	1:400
			AF647	105612	1:400
			Biotin	105604	1:400
CD209b	22D1	eBioscience/ThermoFisher	APC	17-2093-82	1:200
GATA6	D61E4	Cell Signalling Technologies	Purified	5851S	1:800
F4/80	BM8	Biolegend	PE-Cy7	123114	1:200
			APC-Cy7	123118	1:100
FRβ	10/FR2	Biolegend	APC	153306	1:200
			PE	153303	1:200
Sema4a	5E3/SEMA4a	Biolegend	APC	148406	1:100
			PE	148404	1:100
CCR5	HM-CCR5	Biolegend	AF488	107008	1:50
CD62L	MEL-14	Invitrogen	SuperBright 702	67-0621-82	1:100
				11-0621-82	1:100
			FITC		1:100
MHC II (IA-IE)	M5/114.15.2	Biolegend	AF700	107622	1:200
					1:200
			PB	107620	1:200
			APC-Cy7	107628	1:200
Ly6C	HK1.4	Biolegend	BV711	128037	1:400
SiglecF	ES22-10D8	Miltenyi Biotec	Biotin	130-101-861	1:100
VSIG4	NLA14	eBioscience/ThermoFisher	PE-Cy7	25-5752-82	1:200
Ly6G	1A8	Biolegend	Biotin	127604	1:200
			PB	127612	1:200
Tim4	RMT4-54	Biolegend	PE	130006	1:400
			PE-Cy7	130010	1:400
			AF647	130008	1:400
Streptavidin		Biolegend	BV650	405232	1:1000
Zenon anti-rabbit		Molecular Probes	AF647	Z25308	1:200
Siglec F	E50-2440	BD	BV421	562681	1:200
Ki67	REA183	Miltenyi	FITC	130-117-803	1:50
CXCL13	M1/70	Invitrogen	APC	17-7981-82	1:200
CD11c	N418	Biolegend	APC-Cy7	117324	1:200
TNF	MP6-XT22	Biolegend	BV421	506328	1:100

Rat IgG1 Isotype	RTK2071	Biolegend	BV421	400439	1:100
Rabbit IgG	-	ThermoFisher	Purified	I5006	1:200
Anti-Mouse IgM-HRP	14B1	Southern Biotech	HRP	1140-05	1:2000
Anti-Mouse IgG-HRP	-	abcam	HRP	AB6789	1:5000
