# Supplementary material for: Recruited macrophages that colonize the post-inflammatory peritoneal niche convert into functionally divergent resident cells
Source: Nat Commun. 2021 Mar 19;12:1770. doi: 10.1038/s41467-021-21778-0 (PMC7979918; doi:10.1038/s41467-021-21778-0)
Supplement: Supplementary file 3 — Descriptions of Additional Supplementary Files [file 41467_2021_21778_MOESM3_ESM.pdf]

Descriptions of Additional Supplementary Files

Supplementary data 1

Description: Nanostring analysis of RMacZ10 vs IMacZ10 following transfer into native recipient mice

Supplementary data 2

Description: Nanostring analysis of RMac vs RMacZ10 following transfer into native recipient mice

Supplementary data 3

Description: Nanostring analysis of RMacZ10 vs IMacZ10 following transfer into depleted recipient mice

Supplementary data 4

Description: Nanostring analysis of RMac vs RMacZ10 following transfer into depleted recipient mice

Supplementary data 5

Description: Δ Gene rank percentile for each of the detected genes in donor populations transferred into native or depleted recipient
