# Supplementary material for: Recruited macrophages that colonize the post-inflammatory peritoneal niche convert into functionally divergent resident cells
Source: Nat Commun. 2021 Mar 19;12:1770. doi: 10.1038/s41467-021-21778-0 (PMC7979918; doi:10.1038/s41467-021-21778-0)
Supplement: Supplementary file 9 — Reporting Summary [file 41467_2021_21778_MOESM9_ESM.pdf]

nature research

Corresponding author(s): Stephen Jenkins
Last updated by author(s): Jan 24, 2021

Reporting Summary

Nature Research wishes to improve the reproducibility of the work that we publish. This form provides structure for consistency and transparency in reporting. For further information on Nature Research policies, see our Editorial Policies and the Editorial Policy Checklist.

Statistics

For all statistical analyses, confirm that the following items are present in the figure legend, table legend, main text, or Methods section.

n/a	Confirmed	
☐	☒	The exact sample size (*n*) for each experimental group/condition, given as a discrete number and unit of measurement
☐	☒	A statement on whether measurements were taken from distinct samples or whether the same sample was measured repeatedly
☐	☒	The statistical test(s) used AND whether they are one- or two-sided<br>*Only common tests should be described solely by name; describe more complex techniques in the Methods section.*
☒	☐	A description of all covariates tested
☐	☒	A description of any assumptions or corrections, such as tests of normality and adjustment for multiple comparisons
☐	☒	A full description of the statistical parameters including central tendency (e.g. means) or other basic estimates (e.g. regression coefficient) AND variation (e.g. standard deviation) or associated estimates of uncertainty (e.g. confidence intervals)
☐	☒	For null hypothesis testing, the test statistic (e.g. *F*, *t*, *r*) with confidence intervals, effect sizes, degrees of freedom and *P* value noted<br>*Give P values as exact values whenever suitable.*
☒	☐	For Bayesian analysis, information on the choice of priors and Markov chain Monte Carlo settings
☒	☐	For hierarchical and complex designs, identification of the appropriate level for tests and full reporting of outcomes
☒	☐	Estimates of effect sizes (e.g. Cohen's *d*, Pearson's *r*), indicating how they were calculated

Our web collection on statistics for biologists contains articles on many of the points above.

Software and code

Policy information about availability of computer code

Data collection	BD FACS Diva 8.0.3/9.0/ 9.0.1
Data analysis	Flowjo V10.4.1 was used for analysis of flow cytometry datafiles (FCS).<br>Graphpad prism 8 was used to generate graphs and carry out statistical analysis<br>nSolver Version 4 advanced analysis was used to analyze NanoString data.<br>LEGENDplex™ Data Analysis Software (Windows version 8.0) was used to analyse legendplex cytokine data.<br>RStudio Version 1.2 running R version 3.6.1 using the packages GGplot2,Pheatmap,EnhancedVolcano and GOplot was used to generate mRNA data figures and calculate/plot principal components.<br>GSEA 4.1. was used to carry out GSEA analysis.<br>Affinity Designer 1.7.2 was used to generate final combined figures.<br>GEO2R was used for analysis of published datasets (Version info: R 3.2.3, Biobase 2.30.0, GEOquery 2.40.0, limma 3.26.8)

For manuscripts utilizing custom algorithms or software that are central to the research but not yet described in published literature, software must be made available to editors and reviewers. We strongly encourage code deposition in a community repository (e.g. GitHub). See the Nature Research guidelines for submitting code & software for further information.

Data

Policy information about availability of data

All manuscripts must include a data availability statement. This statement should provide the following information, where applicable:

- Accession codes, unique identifiers, or web links for publicly available datasets
- A list of figures that have associated raw data
- A description of any restrictions on data availability

Nanostring data that support the findings of this study have been deposited in the Gene Expression Omnibus under the accession code GSE165036. Previously published publicly available datasets analysed in this study are available in Gene Expression Omnibus under the accession codes GSE56711, GSE37448, GSE47049, and GSE129095. All other data that support the findings of this study are available from the corresponding author upon reasonable request.

Field-specific reporting

Please select the one below that is the best fit for your research. If you are not sure, read the appropriate sections before making your selection.

[x] Life sciences [ ] Behavioural & social sciences [ ] Ecological, evolutionary & environmental sciences

For a reference copy of the document with all sections, see nature.com/documents/nr-reporting-summary-flat.pdf

Life sciences study design

All studies must disclose on these points even when the disclosure is negative.

Sample size

Group sizes used in our animal studies were designed on the basis of trial experiments or the results from the first iteration of each experiment. For time-course experiments the sample size is dictated by the number of cells needed for cell purification at wk8 and consequently groups are larger than would otherwise be required.

Data exclusions

For studies relying on PH26-PCL dye labeling, samples were excluded if no dye staining was detectable in any cells in peritoneal lavage indicating failed IP injection (exclusion criteria determined prior to experiments using non-dye injected controls)

For studies investigating the short term effects of zymosan A, samples were excluded if no monocyte recruitment was apparent indicating failed IP injection of zymosan (exclusion criteria determined prior to experiments).

For clodronate-mediated macrophage depletion studies, recipient mice with >90% Tim4+ resident macrophages within the recipient macrophage compartment were excluded as this indicates a failed IP injection of liposomes leading to the failure to deplete resident cells. These exclusion criteria were determined during development of the depleted 'niche' system and were validated by assessing expression of Tim4 on Kupffer cells, a population that is also depleted by ip injection of clodronate liposomes and on which a lack of Tim4 expression reliably identifies cells recently derived from monocytes (Scott et al, Nature Communications, 2016).

For adoptive transfer studies, any mice in which no donor cells were present were excluded from analysis. Such non-engrafted mice were present in similar numbers across groups and experiments and likely represent a failed IP injection of donor cells, since in all such mice no up-take of transferred dye-label occurred in host cells as shown in Supplementary Figure 3a. (Exclusion criteria determined after the first experimental results).

Animals that were noted as poorly injected at the time of injection, or if the collected peritoneal lavage fluid contained blood or if the gut/peritoneal cavity cells exhibited clear indications of inflammation were excluded from analysis. (Exclusion criteria determined prior to experiments)

One tissue protected bone marrow sample sample was excluded as chimerism was significantly higher than expected based on prior experience in the lab and published work (Bain et al, Nature Communications, 2016). In this sample chimerism of circulating monocytes varied greatly between measurements taken on blood at different time-points making interpretation impossible. (Exclusion criteria established upon analysis)

One sample was excluded from NanoString data analysis as it was flagged up as poor quality. (Exclusion criteria determined following quality control)

Replication

Unless otherwise indicated experiments were carried out independently at least 2 times. All replicate experiments were successful, and data from all replicate experiments were combined for statistical testing and presentation in figures.

Randomization

Animals were randomly assigned treatment groups. For each experiment mice in different treatment groups were co-housed. Studies presented in Figure 1 were carried out across different animal facilities.

Blinding

No blinding was used in our studies since the computation framework was identical for all processed samples.

Reporting for specific materials, systems and methods

We require information from authors about some types of materials, experimental systems and methods used in many studies. Here, indicate whether each material, system or method listed is relevant to your study. If you are not sure if a list item applies to your research, read the appropriate section before selecting a response.

Materials & experimental systems

n/a	Involved in the study	
☐	☒	Antibodies
☒	☐	Eukaryotic cell lines
☒	☐	Palaeontology and archaeology
☐	☒	Animals and other organisms
☒	☐	Human research participants
☒	☐	Clinical data
☒	☐	Dual use research of concern

Methods

n/a	Involved in the study	
☒	☐	ChIP-seq
☐	☒	Flow cytometry
☒	☐	MRI-based neuroimaging

Antibodies

Antibodies used	Details of antibodies used are included in Supplementary Table 2
Validation	All antibodies were from commercial sources and have been validated by the manufacturers, as stated on their associated product pages.

Animals and other organisms

Policy information about studies involving animals; ARRIVE guidelines recommended for reporting animal research

Laboratory animals	The following strains were used in this study:<br>C57BL/6JCrl<br>CD45.1/2 mice (B6.SJL-PtprcaPep3b/BoyJ x C57BL/6JCrl)<br>For some experiments C57BL/6JCrl mice were obtained from Charles river,UK.<br><br>6-10 week old female mice were used through out the study. For each experiment age matched mice were used. Mice obtained from Charles river were allowed to acclimatise for at least 2 weeks prior the experiment. Mice were bred in specific-pathogen free conditions at the University of Edinburgh.<br><br>Animals were housed under the following facility conditions:<br>Temperatures: 19°C - 24°C<br>Humidity: 45-65%<br>Lighting: 12:12 (i.e. 12 hours of light then 12 hours of dark)
Wild animals	No wild animals were used in this study
Field-collected samples	No field collected samples were used in this study
Ethics oversight	Experiments were permitted under license by the UK Home Office and were approved by the University of Edinburgh Animal Welfare and Ethical Review Body.

Note that full information on the approval of the study protocol must also be provided in the manuscript.

Flow Cytometry

Plots

Confirm that:

- ☒ The axis labels state the marker and fluorochrome used (e.g. CD4-FITC).
- ☒ The axis scales are clearly visible. Include numbers along axes only for bottom left plot of group (a 'group' is an analysis of identical markers).
- ☒ All plots are contour plots with outliers or pseudocolor plots.
- ☒ A numerical value for number of cells or percentage (with statistics) is provided.

Methodology

Sample preparation	Preparation is described in the Materials & Methods section of the manuscript

Instrument	BD LSR FORTESSA, BD FACS FUSION, BD FACS ARIA II
Software	Accompanying BD FACS diva software to acquire. FLowjo V10.4.1 to analyze
Cell population abundance	Purity of sorted cell fractions was approx 97% verified by flow cytometry analysis of sorted cell populations. Purity was confirmed during early experiments but due to the time sensitivity of subsequent experiments and the low numbers of cells was not done for each subsequent experiment.
Gating strategy	The full gating strategy is indicated in the relevant figures. Gates were set on the basis of FMO or isotype controls

☒ Tick this box to confirm that a figure exemplifying the gating strategy is provided in the Supplementary Information.
